# Bacterial Infection among Cancer Patients: Analysis of Isolates and Antibiotic Sensitivity Pattern

**DOI:** 10.1155/2021/8883700

**Published:** 2021-01-07

**Authors:** Sevitha Bhat, Shruthi Muthunatarajan, Shalini Shenoy Mulki, K. Archana Bhat, K. Himani Kotian

**Affiliations:** ^1^Department of Microbiology, Kasturba Medical College Mangalore, Manipal Academy of Higher Education, Manipal, Karnataka, India; ^2^Kasturba Medical College Mangalore, Manipal Academy of Higher Education, Manipal, Karnataka, India; ^3^Department of Community Medicine, Kasturba Medical College Mangalore, Manipal Academy of Higher Education, Manipal, Karnataka, India

## Abstract

**Introduction:**

Cancer patients being immunosuppressed are vulnerable to develop infections. Knowledge of the changing epidemiology of infections has a pivotal role in its management. *Aims and Objectives*. The study is undertaken to assess the types of bacterial infections in cancer patients undergoing anticancer treatment, the associated bacterial pathogens, and their antibiotic sensitivity patterns.

**Materials and Methods:**

A retrospective surveillance study was undertaken in our center. Positive culture reports and other clinical details of cancer patients diagnosed with infection during a stay in the tertiary care center from 1st January 2015 to 31st December 2016 were analysed by descriptive statistical methods chi-square test and odds ratio to study the association.

**Results:**

Out of 638 cancer patients diagnosed with infections in the 2-year period, 140 patients had positive cultures, representing 272 specimens and 306 isolates. Common specimens sent for culture were blood sputum, urine, and pus. 214 isolates (69.9%) were gram-negative bacilli, and 92 (30.1%) were gram-positive cocci. The most common isolates were *Klebsiella* spp. (18.30%), *Pseudomonas* spp. (17.65%), and *Escherichia coli* (14.71%) followed by *Staphylococcus aureus* (13.72%). Among the gram-negative organisms, the antibiotic resistance rates reported to fluoroquinolones, aminoglycosides, and third-generation cephalosporins were 45.13%, 39.20%, and 48.58%, respectively. 26.92% of the organisms are resistant to all three antibiotics. 50.4% of *Klebsiella* spp. and *Escherichia coli* were ESBL producers. Gram-negative organisms showed 11.63% resistance to *β*-lactam/*β*-lactamase inhibitor combination, and 22.22% of gram-negative organisms are resistant to carbapenems. 50% of the *Staphylococcus* spp. were methicillin resistant, but all were sensitive to vancomycin.

**Conclusion:**

The surge in the number of gram-negative infections emphasizes the need for broad-spectrum empirical therapy targeting the same. Rate of resistance of the isolated gram-negative organisms to the routinely used empirical therapy is alarming. Prudent use of antibiotics, based on culture reports wherever possible, is of utmost importance to save the lives of infected patients and prevent further development of antibiotic resistance.

## 1. Introduction

It is unmistakable that patients in Oncology wards are more vulnerable to develop infections. Cancer and chemotherapy predispose these patients to infection [1]. Infection is commonly encountered among cancer patients, leading to disturbances in the treatment regimen, prolonged hospitalization, increased cost of health care, and reduced survival.

Whilst the mortality rates have fallen over the past years, infection remains a primary or associated cause of death, with bacteria most commonly accounting for infection-associated mortality, followed by fungi [2]. The management of the infections is based on the use of appropriate empirical antimicrobial therapy with a comprehensive understanding of the commonly encountered pathogens and antibiotic sensitivity patterns. Due to this, the relative incidence of gram-negative bacterial infections has declined over the past 20 years, but gram-positive bacteria are more commonly seen [3]. The empirical use of antimicrobials has reduced the mortality in patients but has also led to the menace of multidrug-resistant bacteria [4]. Multidrug-resistant bacteria are commonly encountered among immunocompromised patients.

To successfully prevent, identify, and treat infections, sound knowledge of the ever-changing spectrum of infections is necessary. Management of infections is a major challenge [5]. This present study aims to evaluate the common types of infections in cancer patients undergoing various forms of treatment, the associated bacterial pathogens, and their antibiotic susceptibility pattern. The understanding will aid in personalizing treatment, improving prognosis, and reducing the cost of health care.

## 2. Aims and Objectives

The study was undertaken to monitor the types of the bacterial infections seen in cancer patients undergoing anticancer treatment, the associated bacterial pathogens with their antibiotic sensitivity patterns, types of infections associated with the type of cancers, and modes of treatment like chemotherapy and radiotherapy.

## 3. Materials and Methods

A retrospective surveillance study was conducted in the Department of Oncology of Kasturba Medical College Hospital (KMCHA), Attavar, Mangalore, and the Department of Clinical Microbiology of Kasturba Medical College (KMC), Mangalore. Inclusion criteria were all the patients admitted to the hospital for the treatment of cancer and diagnosed with infection during the period from 1st January 2015 to 31st December 2016.

Microbiological investigations: the clinical specimen received from suspected cases of infection were stained with Gram stain inoculated onto blood agar, chocolate agar, and MacConkey agar (HiMedia) and incubated aerobically at 35°C for 18 hours. Blood culture was done by BacT/ALERT system (BioMerieux, USA). Positive cultures were subcultured onto blood agar, chocolate agar, and MacConkey agar (HiMedia) and incubated aerobically at 35°C for 18 hours. The identification of the bacterial growth and antimicrobial susceptibility testing of the isolates was performed using the VITEK 2 system (BioMerieux, France), and minimum inhibitory concentration (MIC) was interpreted as sensitive or resistant using the Clinical and Laboratory Standards Institute (CLSI) guidelines. The antibiotics tested in VITEK 2 system included amikacin, amoxyclav, ampicillin, cefixime, ceftazidime, ceftriaxone, ciprofloxacin, ertapenem, imipenem, meropenem, gentamicin, piperacillin tazobactam, and trimethoprim-sulfamethoxazole for gram-negative organisms. The antibiotics for gram-positive cocci were cefoxitin, cefalothin, cefoperazone, gentamicin, erythromycin, clindamycin, chloramphenicol, rifampicin, netillin, linezolid, teicoplanin, and vancomycin.

The demographic and clinical data of the patients were collected from the case files including information on age, sex, type of cancer, type of treatment, type of infection, type of bacterial isolate, antibiotic sensitivity pattern, and details: anthropometry, comorbidities, haematological examination results, and any procedures (urinary catheterization, central or peripheral IV cannulation, endotracheal intubation, and ventilator management).

The collected data were entered into Microsoft Excel and analysed by descriptive statistical methods. The association was established by chi-square test and odds ratio. The results obtained are represented in the form of graphs and frequency tables.

## 4. Results

A total of 3784 patients were admitted to KMCHA for the treatment of cancer during the study period. 638 persons were diagnosed with infections. Out of these, 140 patients had documented infections with culture-positive isolates.

**Figure 1:**
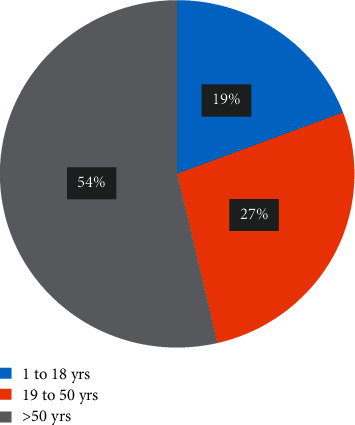
Age-wise distribution of the cancer patients with documented bacterial infections.

**Figure 2:**
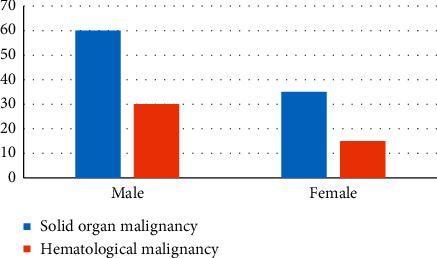
Gender-wise distribution of the cancer patients with documented bacterial infections.

The age and sex distribution are shown in Figures 1 and 2, respectively. The average age of patients with solid organ tumours was 52 years. The maximum number of cases belonged to the age group of 50–55 years. The oldest patient was 80-year-old and the youngest 2 years of age. The average age of patients with haematological malignancy was 30 years. The maximum number of patients belonged to the age group 10–15 years. The oldest patient was 72-year-old, and the youngest was 1-year-old.

The most commonly encountered type of clinical infection was bloodstream infection (33.33%) in patients with haematological malignancies and respiratory tract infections (34.93%) in patients with solid organ tumours (Table 1).

**Figure 3:**
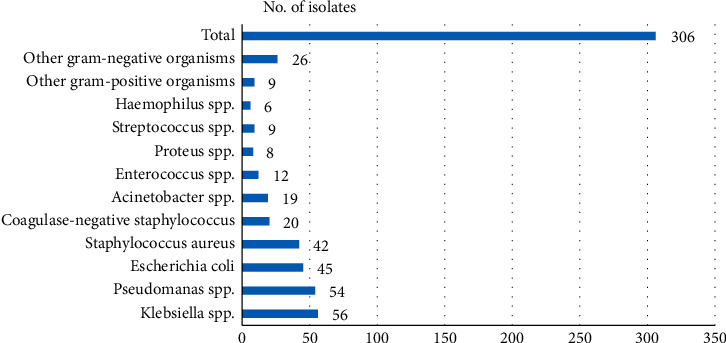
Distribution of bacterial isolates among cancer patients.

The etiological agents associated with infections are shown in Table 2. From the 140 patients mentioned above, 272 specimens were culture positive, from which 306 microorganisms were isolated. The most common isolate was *Klebsiella* spp. (18.30%). 214 isolates (69.93%) were gram-negative bacilli, and 92 (30.07%) were gram-positive cocci (Figure 3).

The most common isolate associated with bloodstream infections was *Klebsiella* spp. *Pseudomonas* spp. was commonly seen in respiratory tract infection, *Escherichia coli* in urinary tract infections, and *Staphylococcus aureus* in the skin and soft tissue infections.

The antibiotic resistance patterns of gram-negative and gram-positive isolates are shown in Tables 3 and 4, respectively.

The association of neutropenia with the outcome is shown in Table 5. Since *p* value is >0.05, there is no association between neutropenia and mortality. The risk of mortality was 1.22 times higher in neutropenic patients compared to nonneutropenic patients (odd's ratio 1.224).

**Figure 4:**
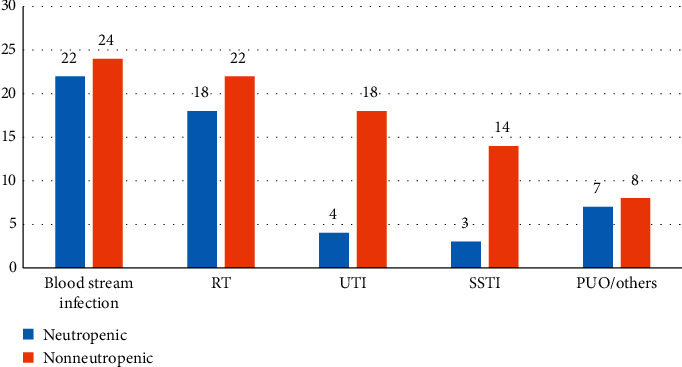
Distribution of infection sites in neutropenic and nonneutropenic patients.

The infection sites in neutropenic and nonneutropenic patients are depicted in Figure 4.

80% and 27.9% of the 140 patients were on chemotherapy and radiotherapy, respectively, and 21.4% underwent surgical procedures. The overall mortality rate in cancer patients with documented infection was 60%.

## 5. Discussion

Infections are still a cause of substantial morbidity and mortality in cancer patients. In our settings, mortality was observed in 60% of the 140 cancer patients with microbiologically proven infections, and the rest caused significant morbidity and the increased expense of patient care. The important infections like bloodstream infections and pneumonia were major contributors to mortality in oncology patients. The previous studies have reported 36% mortality due to sepsis in cancer patients [6]. Pneumonia, sepsis, influenza, and parasitic infections have been documented among the deceased cancer patients [7].

In our study, out of the 306 isolated organisms, 214 were gram-negative and 92 were gram-positive bacteria. 69.9% of the infections were associated with gram-negative organisms. This is in contrast to the earlier reports from developed countries, where the incidence of infections caused by gram-positive bacteria is higher. In most of the studies from developed countries, around 70% of the infections are caused by gram-positive bacteria [5]. On the contrary, most studies conducted in developing countries have recorded that majority of infections were caused by gram-negative organisms [8–10]. The explanation to this fact may be attributed to the reduced use of indwelling catheters and devices and less usage of prophylactic antimicrobial regimens in neutropenic patients in different setups.

Epidemiology of infections in cancer patients has changed across the globe overtime and is characterized by a shift from gram-negative bacteria (1960s and 1970s) to gram-positive ones (1980s). Gram-negative bacteria have predominated the scene as a major cause of infections in cancer patients in the last 20 years across the globe in many countries.

Among gram-negative bacteria, *Klebsiella pneumoniae, Pseudomonas aeruginosa*, and *Acinetobacter baumannii* have been increasingly associated with cancer patients. However, the frequency of occurrence of such MDR-resistant organisms is variable depending on the reporting country and continent. At the same time, inadequate empirical/therapeutic therapy with antibiotics exposes these patients to increased risk of adverse outcome, especially in neutropenic bacteremic patients suffering from MDR infections [11].

The findings of a study conducted by Yadegarynia et al. in Texas showed that pneumonia was the most common infection seen in both the groups of patients with solid organ tumours (26%), as well as in patients with haematological malignancies (38%) [12] Bloodstream infections were the second most common in patients with haematological malignancies (35%) and urinary tract infections in patients with solid organ tumours (22%). This is in contrast to results from our study, where bloodstream infections (32.8%) were more common than respiratory tract infections (28.57%). Bloodstream infections (33.33%) were more common in haematological malignancies, followed by respiratory tract infections (19.2%). In patients with solid organ tumours, respiratory tract infections (34.93%) were commonly seen, followed by bloodstream infections (32.53%). The study has reported a surge in the incidence of polymicrobial infections (23%–31%). Our study differs in this, as only 35 of the 272 samples (12.9%) isolated multiple organisms [13].

In a study conducted by Kumar et al. in Mumbai, the overall rank order of the most common pathogens was *Pseudomonas* spp. (26.2%) > *Enterococcus* spp. (11.66%) > *S. aureus* (11.44%) > *E. coli* (11.34%) > *Klebsiella* spp. (10.59%) > *Acinetobacter* spp. (9.95%) > Coagulase-negative S*taphylococcus* (CoNS) (6.52%) > *Streptococcus* spp. (3.42%) > *Enterobacter* spp. (3.1%) > *Burkholderia* spp. (2.35%) [14]. This was not the case in our study, where rank order was *Klebsiella* spp. (18.30%) > *Pseudomonas* spp. (17.65%) > *Escherichia coli* (14.71%) > *Staphylococcus aureus* (13.72%) > Coagulase-negative *Staphylococcus* (6.54%) > *Acinetobacter* spp. (6.21%) > *Enterococcus* spp. (3.92%) > *Proteus* spp. (2.61%) > *Streptococcus* spp. (2.94%) > *Haemophilus* spp. (1.96%). They found gram negative accounted for 66.96% of the isolates, which is similar to our findings of 69.9% [14].

*Acinetobacter* spp, a nonlactose fermenting, multidrug-resistant organism, known to be a significant bug among neutropenic patients, accounted for 6.21% of the isolates. This is less than findings of Kumar et al. (9.95%) but greater than the findings of Siddaiahgari et al. in a study conducted in Hyderabad, among pediatric patients, who found only two out of 89 isolates (2.2%) were *Acinetobacter* spp. [14].

In the study conducted by Siddaiahgari et al., *Pseudomonas* spp. was the most common causative organism of bloodstream infection, causing 36% of the bloodstream infections. *E. coli* accounted for 46.3% of the urinary tract infections [14]. In our study, *Klebsiella* spp. was most common, causing 21.2% of the bloodstream infection. Similar to their study, *E. coli* was the predominant etiological agent of UTI in our study, causing 63.2% of the infections [15].

A study noted that 68.18% of the isolated from blood stream infections were gram negative, which is similar to the findings of our study. The same study also noted that there was low occurrence (18%) of methicillin-resistant *S. aureus*, whereas our study showed that 41% of *S. aureus* were methicillin resistant. They noted that 50% of Enterococcus were vancomycin resistant, whereas, in our study, only 8.3% of Enterococcus were vancomycin-resistant enterococci (VRE). This may be attributed to the fact that vancomycin is not used for empirical prophylactic therapy in our hospital settings [16].

Analysis of antibiotic resistance of gram-negative organisms revealed 50.4% of the isolates were ESBL producers, which is less than what is commonly seen in other studies. A study done in New Delhi by Batra et al. noted 80% ESBL production rates among the gram-negative bugs. ^16^Carbapenem resistance in our study was noted to be 15.4% among *Klebsiella* spp. and 17% among Pseudomonas spp. which is more promising than the results of a study, where 49% of *Klebsiella* spp. and 31% of *Pseudomonas* spp. were resistant. In contrast, *E. coli* in our study (15.6%) showed more carbapenem resistance than theirs (11%). Fluoroquinolone and aminoglycoside resistance in gram-negative isolates was noted to be 45.6% and 37.3%, respectively, in contrast to the higher resistance of about 70% and 64.7%, respectively, seen in the study done in Mumbai.

In the study by Eleni Isidora et al., 14% of the study group had previous MDR colonization, which possibly contributes an increased risk of MDR infection. We have not studied the colonization of MDR bacteria in the cancer patients.

A systematic review and meta-analysis revealed high colonization with extended beta-lactamase producing Enterobacteriaceae among patients with solid or haematological malignancy. This phenomenon increases the risk of bacteremia with the same pathogen and created important reservoirs for horizontal spread between oncological hospitalized patients [17].

In our study, chemotherapy was the most common mode of treatment with 80% of patients undergoing chemotherapy.

The risk of mortality was 1.22 times higher in neutropenic patients compared to nonneutropenic patients (odd's ratio 1.224). The results of a study among 7512 critically ill cancer patients confirmed neutropenia to be independently associated with mortality [18]. Febrile neutropenia is a challenge to deal with in case of haematological malignancies. There is a pivotal role of empiric antibiotics in this scenario to prolong the longevity.

A systematic review showed that neutropenia was associated with a 10% rise in overall mortality [19].

84 of the cancer patients succumbed to infections. Among the 84 patients, 71 (84%) cancer patients with documented bacterial infections had infections with MDR *Klebsiella pneumoniae* and *Acinetobacter baumannii*. MDR bacterial infections were thus significantly associated with mortality in cancer patients [13].

Limitations of our study include its retrospective design and single institutional nature. Our study included only the positive cultures of those patients in whom infection was suspected and not of all cancer patients.

## 6. Conclusion

Despite the improved management of cancer patients, infections remain a significant cause of mortality among cancer patients. Implementation of strict infection control practices would go a long way in improving this dreaded situation. In developing countries, the broad-spectrum empirical therapy provided must focus more on the treatment of gram-negative infections. However, not only does it increase the cost of patient care but also leads to the selection of multidrug-resistant organisms, as seen abundantly in this study, as well as many others. The prospective studies on the antibiotic sensitivity patterns in hospitals will help to formulate local guidelines and therapeutic strategies. The increasing development of resistance to existing antimicrobials necessitates a dire need to develop novel agents at a rate faster than the development of resistance. It is of utmost importance to restrict the use of antibiotics in all clinical practices, using narrow-spectrum antibiotics based on culture reports wherever possible. This may come a long way in improving the situation of patients with life-threatening infections, especially in those who are immunocompromised.

## 7. Summary

Infections among cancer patients are a major challenge to deal with. They cause suboptimal delivery of chemotherapy which leads to poor treatment outcome, adds to cost of management, and contributes to increased morbidity. Resistant organisms have emerged owing to selective antimicrobial pressure, which further complicates the problem. To successfully prevent, identify, and treat infections, knowledge of the changing epidemiology of infections is essential. In this study, we examined the types of bacterial infections seen in cancer patients undergoing anticancer treatment, the associated bacterial pathogens, and their antibiotic sensitivity patterns:

1. The study included 140 cancer patients who were diagnosed clinically with infection and showed culture positivity. 182 infections were encountered in these patients, 272 specimens obtained from these patients were culture positive, and 306 organisms were isolated.
2. The percentage of deaths in cancer patients with infections was found to be 60%. It was greater in those with solid organ tumours than in those with haematological malignancies.
3. Bloodstream infections accounted for 36.3% of the total infections. Second most common were respiratory tract infections, accounting for 31.9%.
4. Chemotherapy was the most common mode of treatment.
5. 69.9% isolates were gram negative and 30.1% were gram positive. Most common isolate was *Klebsiella* spp. (18.30%).
6. Among the gram-negative organisms, 45.13% of the organisms tested showed resistance to fluoroquinolones, 39.20% show resistance to aminoglycosides, 48.58% show resistance to third-generation cephalosporins, and 26.92% of the organisms are resistant to all three antibiotics.
7. 51.8% of the isolated *Klebsiella* spp. and 48.9% of the isolated *Escherichia coli* were ESBL producers.
8. Gram-negative organisms show 11.63% resistance to *β*-lactam/*β*-lactamase inhibitor combination, and 22.22% of gram-negative organisms are resistant to carbapenems.
9. 41% of *S. aureus* were methicillin resistant. All the MRSA were susceptible to vancomycin.
10. 8.3% of *Enterococcus* spp. were resistant to vancomycin.
11. Implementing strict infection control practices, conducting frequent prospective studies on the antibiotic sensitivity patterns seen in hospitals to help formulate local guidelines, developing novel antimicrobial agents, and restricting the use of antibiotics in clinical practices is important to reduce the incidence and improve the prognosis of infections in cancer patients.

## 8. Study Implications

This study helps to achieve a precise knowledge of the common types of infections seen in cancer patients undergoing various forms of therapy, the associated bacterial isolates, and their antibiotic susceptibility. This understanding will aid in formulating a personalised and cost-effective treatment, improving prognosis, and ensuring the prudent use of antibiotics.

## Figures and Tables

**Table 1:** Types of infection in patients with solid tumors and haematological malignancies.

Types of Infection	No. of infections (%)		
	In patients with solid tumours (*N* = 83)	In patients with haematological malignancies (*N* = 57)	Patients with infection (*N* = 140)
Bloodstream	27 (32.53%)	19 (33.33%)	46 (32.8%)
Respiratory tract	29 (34.93%)	11 (19.2%)	40 (28.57%)
Urinary tract	16 (19.2%)	6 (10.5%)	22 (15.71%)
Skin and soft tissue	4 (9.3%)	13 (22.8%)	17 (12.14%)
Others (PUO, GI)	7 (8.4%)	8 (14%)	15 (10.71%)

**Table 2:** Types of infections caused by various organisms.

Organism	Types of infection				
	Bloodstream	Respiratory tract	Urinary tract	Skin and soft tissue	Others
*Klebsiella* spp.	22	18	9	5	2
*Pseudomonas* spp.	10	24	9	11	0
*Escherichia coli*	21	1	18	4	1
*Staphylococcus aureus*	13	2	2	22	3

**Table 3:** Antibiotic resistance pattern of gram-negative isolates.

	Quinolone resistance	Aminoglycoside resistance	ESBL	Carbapenem resistance
*Klebsiella* spp.	19/50 (38.0%)	21/54 (38.9%)	29/56 (51.8%)	8/52 (15.4%)
*Pseudomonas* spp.	17/50 (34.0%)	13/50 (26.0%)		9/53 (17.0%)
*Escherichia coli*	34/41 (82.9%)	18/42 (42.8%)	22/45 (48.9%)	5/32 (15.6%)

**Table 4:** Antibiotic resistance pattern of gram-positive isolates.

	Methicillin resistance	Vancomycin resistance	Macrolide resistance	Aminoglycoside resistance
*Staphylococcus aureus*	16/39 (41.0%)	0	29/42 (69.0%)	11/39 (28.2%)
*Coagulase-negative Staphylococcus*	11/15 (73.3%)	0	13/19 (68.4%)	3/19 (15.8%)
*Enterococcus* spp.	—	1/12 (8.3%)	1/5 (2.0%)	8/11 (72.7%)

**Table 5:** Association of neutropenia with outcome (% is within neutropenic and nonneutropenic episodes).

Outcome	Neutropenic	Nonneutropenic	Chi-square test	*p* value
Dead	34 (63%)	50 (58.1%)	0.322	0.571
Stable	20 (37%)	36 (41.9)		
Total	54	86		

## Data Availability

All datasets generated or analysed during this study are included in the manuscript and the supplementary files.

## Supplementary Materials

Supplementary Materials The patient clinical and laboratory details are included in the database provided.
